# Capacitive sensor based on molecularly imprinted polymers for detection of the insecticide imidacloprid in water

**DOI:** 10.1038/s41598-020-71325-y

**Published:** 2020-09-02

**Authors:** Suzan El-Akaad, Mona A. Mohamed, Nada S. Abdelwahab, Eglal A. Abdelaleem, Sarah De Saeger, Natalia Beloglazova

**Affiliations:** 1 Centre of Excellence in Mycotoxicology and Public Health, Faculty of Pharmaceutical Sciences, Ghent University, Ghent, Belgium; 2 Department of Pharmaceutical Chemistry, National Organization for Drug Control and Research (NODCAR), Giza, Egypt; 3 Department of Analytical Chemistry, Faculty of Pharmaceutical Sciences, Beni-Suef University, Benisuef, Egypt; 4 Nanotechnology Education and Research Center, South Ural State University, Chelyabinsk, Russia; 5 Department of General and Inorganic Chemistry, Chemistry Institute, Saratov State University, Astrakhanskaya, Saratov, Russia

**Keywords:** Sensors and biosensors, Lab-on-a-chip, Sensors

## Abstract

This manuscript reports on the development of a capacitive sensor for the detection of imidacloprid (IMD) in water samples based on molecularly imprinted polymers (MIPs). MIPs used as recognition elements were synthesized via a photo-initiated emulsion polymerization. The particles were carefully washed using a methanol (MeOH) /acetic acid mixture to ensure complete template removal and were then dried. The average size of the obtained particles was less than 1 µm. The imprinting factor (IF) for IMD was 6 and the selectivity factor (α) for acetamiprid, clothianidin, thiacloprid and thiamethoxam were 14.8, 6.8, 7.1 and 8.2, respectively. The particles were immobilized on the surface of a gold electrode by electropolymerization. The immobilized electrode could be spontaneously regenerated using a mixture of MeOH/10 mM of phosphate buffer (pH = 7.2)/triethylamine before each measurement and could be reused for 32 times. This is the first-time that automated regeneration was introduced as part of a sensing platform for IMD detection. The developed sensor was validated by the analysis of artificially spiked water samples. Under the optimal conditions, the linearity was in the range of 5–100 µM, with a limit of detection (LOD) of 4.61 µM.

## Introduction

Nowadays, neonicotinoids (neonics) are the most important class of pesticides in the global market. Their “success” story started in 1991 by introducing imidacloprid (IMD) on the market. Since then, for many years IMD has become the world’s largest selling pesticide with the registered uses for over 140 crops in 120 countries^1,2^. It is extensively used at a large scale with applications ranging from plant protection, veterinary products, and seed coating. As a result of its extensive usage, IMD can be found as a pollutant in all environmental compartments (soil, water and air)^3^. Neonics act by binding to the nicotinic acetylcholine receptors (nAChRs) in their target invertebrates. They mimic the action of neurotransmitters, leading to the continuous stimulation of neurons. In doing so, they could cause the death of their target invertebrates^3^. Neonics are highly water soluble, persistent in water, soil, minimally degraded by light and not volatile, which make them easily transported from an area of application to different environmental compartments^4^. In addition to that, it was found that like other pesticides they have a negative impact on non-target organisms. IMD and other members of the first generation are highly toxic to bees^5,6^. These chemical properties in addition to their negative environmental impacts have raised EU concern^7^.

Pesticides in general could easily contaminate supplies of drinking water via surface or ground water systems. Depending on the quantity and toxicity level of a pesticide together with the frequency of exposure to the contaminated drinking water, pesticides in water could negatively affect human health and environment. It was found that neonics could have lethal and sublethal effects on many aquatic invertebrates. The acute and chronic neonics toxicity differs significantly among aquatic arthropods (the LC50 values range from < 1 to > 100,000 μg/L). Although this class of pesticides is highly controversial, there are few quality reference values for neonics in surface waters. The actual guidelines on ecological water quality differ extensively from country to country, and many of them are still under review^8^.

Due to the extensive use of neonics, it became very important to develop rapid and reliable techniques for their detection and quantification, in different matrices. Many chromatographic methods have been developed to monitor trace levels of neonics^9,10^. Although those methods are accurate, they are time consuming, not applicable for on-site performance, they require experienced personnel and expensive equipment, sophisticated sample preparation and high amounts of “toxic” organic solvents. Therefore, electrochemical methods have emerged as a promising alternative technique. Electrochemical sensors are robust, easy-in-use, feasible for on-site application and demonstrate high sensitivities^11–14^. To enhance their selectivity, recognition elements can be introduced.

Molecularly imprinted polymers (MIPs) possess selective recognition sites that are complimentary in shapes, charges, and functionalities to the chosen targets. They could be synthesized by a thermo-, photo-, or electrochemically-initiated polymerization^15^. MIPs’ excellent high thermal and chemical stability and selectivity made them a promising alternative to natural bioreceptors for analysis in complex matrices. MIPs have been widely used in chromatography^10^, solid-phase extraction (SPE)^16^, drug delivery^17^, water treatment^18^, membrane separations^19^, and chemical sensors^20,21^. MIPs have been used as receptors in many electrochemical platforms. MIP-based electrochemical detection of herbicides^22^ pesticides^23^, insecticides^24,25^ and fungicides^26^ has been quite intensively published.

There have been some reports on the application of MIPs for the detection of IMD in different matrices (Table S1). Nearly all the previously synthesized MIPs toward IMD were primarily used for SPE and focused mainly on foodstuffs^10,16,27,28^. However, there are a few reports on the application of MIPs in electrochemical sensing for IMD detection in vegetables^20,29^, fruits^29,30^, rice^31^, celery juice^32^ and water samples^33^. However, those sensors show a low limit of detection, and removal of the template after polymerisation is a general drawback for in-situ-synthesized MIPs-based sensors^34^. For label-free sensors this leakage can potentially lead to false measurements. However, this type of sensors cannot be subjected to any harsh conditions for template removal as this procedure could possibly affect the adsorption-based surface of the functionalized electrode. Besides, the published MIPs-based sensors for IMD lack regeneration, and thus their reusability, which is a serious limitation for any on-site application. The existing developed immunosensors based on antibody as a recognition element were not validated for ground or river water neither for irrigation systems knowing that an antibody is not stable in harsh environments and therefore not reliable for on-line sensing of pesticides^35^.

In this paper, a capacitive MIP-based sensor for IMD detection was proposed. MIPs were obtained using a facile and fast synthesis method (1 h). After the synthesis, the obtained MIP-beads were attached to an electrode surface. By this two-step procedure (synthesis and immobilization) damage of the electrodes could be avoided, and the absence of any remaining template molecules in the obtained particles attached on the electrode was ensured.

Capacitive sensors fall into the category of impedance sensors. Due to their superior robustness, simplicity and sensitivity, capacitive sensors became very interesting in recent years. Different analytes were detected using capacitive sensors with a high sensitivity, selectivity and low sample volumes primarily in medical, biomedical and biological applications^36–39^. The measuring unit is in (nF) due to the high stability of the baseline^34^. In our work, we present a novel capacitive sensor based on MIPs for the rapid and label-free detection of IMD in water samples. To the best of our knowledge, this is the first capacitive sensor based on MIPs for the detection of IMD in water. Moreover, for the first time, a two-step approach with a regeneration step between each analysis was introduced for IMD sensing, adding the possibility to the sequential use of each electrode for 32 times in which real water samples were used for the validation of the system.

## Materials and methods

### Materials

Azobisisobutyronitrile (AIBN, 98%), imidacloprid PESTANAL analytical standard (IMD) ethylene glycol dimethacrylate (EGDMA, 98%), hydrogen peroxide (30 wt%), methacrylic acid (MAA, 99%), tyramine (99%), dipotassium hydrogen phosphate (K_2_HPO_4_, ≥ 98%), 1-dodecanethiol (≥ 98%), trimethylamine (≥ 99%), sodium dodecyl sulphate (SDS) and potassium ferricyanide (K_3_[Fe (CN)_6_]) were purchased from Sigma Aldrich (Bornem, Belgium). Methanol (MeOH, LC–MS grade) and acetonitrile (ACN, LC–MS grade) were purchased from Biosolve BV (Valkenswaard, Netherlands). Acetone (99.5%) was obtained from Fiers (Kuurne, Belgium) and ethanol (EtOH absolute, Analar Normapure) from VWR International (Leuven, Belgium). Ultrapure water was obtained with the ultra-pure water system from arium pro, Sartorius (Goettingen, Germany). Gold electrodes were provided by CapSenze AB (Lund, Sweden). A 25 mL quartz glass round flask was purchased from Witeg Labortechnik GmbH (Wertheim, Germany). All the electrochemical measurements were carried out using a PGSTAT 101 potentiostat (Metrohm, Utrecht, The Netherlands) coupled to a computer using NOVA software (version 2.0) for data acquisition. An automated flow injection capacitance system based on current pulse capacitive measurements was used to perform the analysis (CapSenze HB, Lund, Sweden).

### Synthesis of the polymers

**Figure 1:**
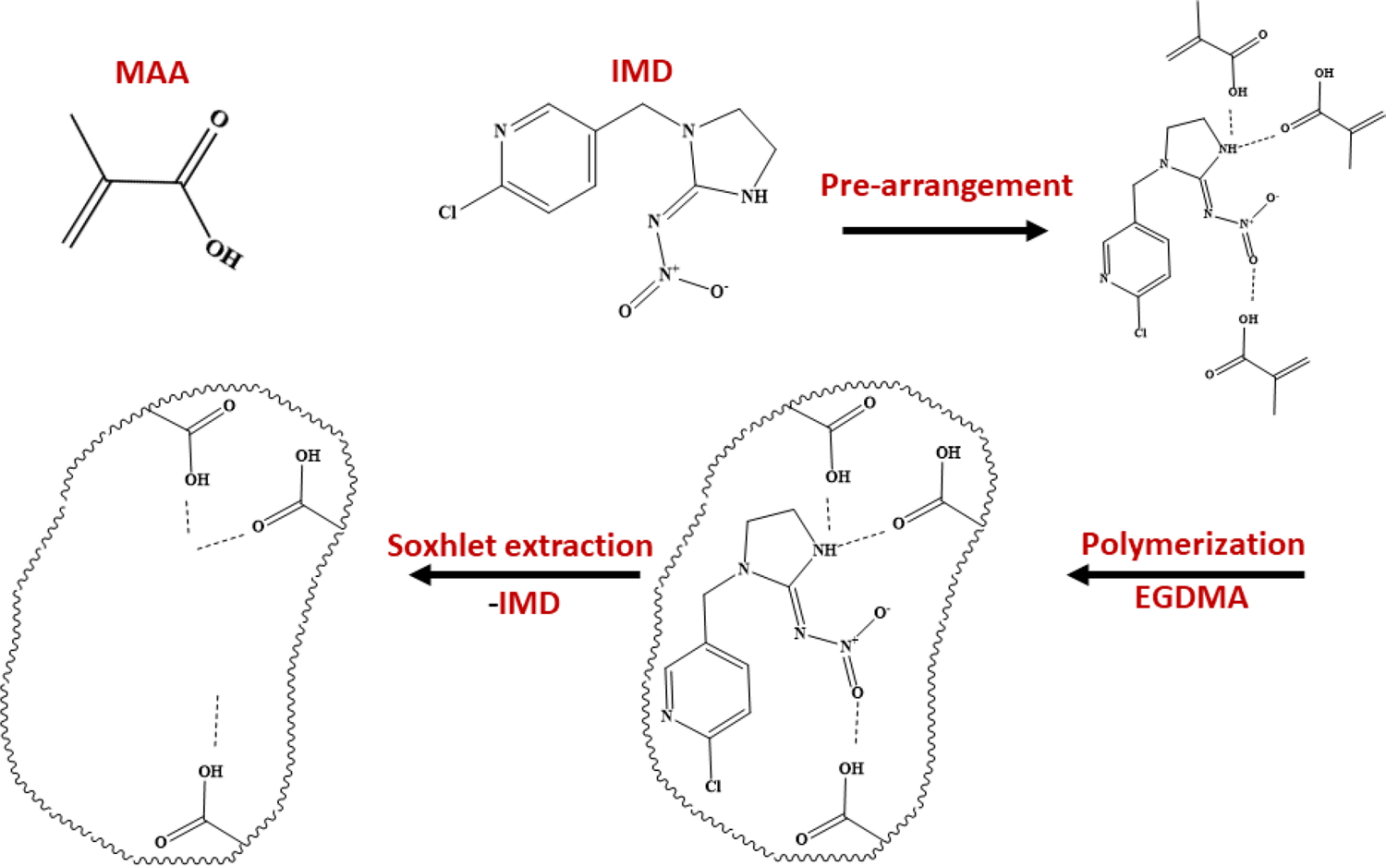
Schematic illustration of the IMD molecular imprinting procedure.

The IMD-selective MIPs were prepared by emulsion polymerization. IMD was used as the template, MAA—as the monomer and EGDMA—as the cross-linker with a molar ratio of template/monomer/cross-linker of 1:4:20. First of all, 0.1 mmol of the template (IMD) were dissolved in 0.95 mL of ACN in a 10 mL-long glass flask. Then, 0.4 mmol of the functional monomer (MAA) were added and incubated with the template for 1 h. After this, 1.6 mmol of the crosslinker (EGDMA), 22 mg of the initiator (AIBN), and 19.33 µL of hexadecane were added and stirred at 500 rpm with a magnetic stirrer. After that, 5 mL of the surfactant solution (0.1 M SDS) were added and the content was homogenized at 12,000 rpm for one minute using a T25 digital ULTRA-TURRAX disperser (IKA, Staufen, Germany). The obtained emulsion was transferred to a 25 mL-quartz glass round flask with a magnetic stirrer, and a tap was placed on the flask. Nitrogen was introduced into the flask for 15 min. Next, the flask was placed 20 cm away from the UV light (75 mW/cm^2^; λ = 365 nm), and the polymerization reaction was initiated and continued for 1 h. After the polymerization, the mixture was collected and left to dry at room temperature. After the drying, the polymer particles were collected and washed with Soxhlet in a MeOH/acetic acid mixture (95/5, v/v) (Fig. 1). The average size of the particles was measured using a Zetasizer Nano ZS (Malvern Instruments Ltd., Worcestershire, UK). The non-imprinted polymers (NIPs) were prepared in the same manner, but without adding the template.

### Coupling of the polymer beads to a gold electrode surface

A gold electrode was cleaned to remove any coatings from its surface. For this, the electrode was submerged in acetone, ethanol and piranha solution (H_2_SO_4_:H_2_O_2_; 3:1), successively, for 10 min each and then dried with nitrogen. From the MIP or NIP powder, 2.5 mg was suspended in a 10 mM tyramine solution by sonication. The electrode was fixed in a reaction cell, and 300 µL of the suspended solution were added on the electrode active surface. The particles were allowed to sediment for 15–20 min before the electropolymerization step. A polytyramine layer was formed after 15 potential sweeps between 0 and 1.5 V with a scan rate of 50 mV s^−1^. Finally, any remaining bare sites on the gold electrode surface were blocked by placing the electrode in a 10 mM 1-dodecanthiol in ethanol solution for 20 min. Cyclic voltammetry was used to verify that each layer was properly formed by checking the redox peak currents.

### Automated flow injection system

An automated flow injection system developed by Capsenze HB (Lund, Sweden) was used to perform the measurements. This system was described by Erlandsson^40^. It resembles a flowing water body as a river or a continuous stream in a laboratory environment. A running buffer [10 mM KH_2_PO_4_/K_2_HPO_4_ buffer (pH = 7.2)] carried the standards, samples or the regeneration buffer [MeOH/10 mM KH_2_PO4/K_2_HPO_4_ buffer (pH = 7.2)/triethylamine (47.5/47.5/5, v/v/v)] from the pump to the electrochemical flow cell. Following each measurement, the regeneration buffer was applied to interrupt the analyte/MIP complex. This allows the reusing of the electrode for several times (up to 32 times). The MIP or NIP-modified electrode was placed in an electrochemical flow-cell fitted with two platinum wires acting as the auxiliary and the reference electrodes and the capacitance measurements were performed. The measurement was carried out with a steady current of ± 10 µA using the current step method. Every 60 s a pulse was supplied. The capacitance was measured and quantified based on the resulting documented potential profile. The binding event between the analyte and the immobilized MIPs resulted in a decrease in the capacitance value. The capacitance values were recorded with a flow rate of 1.67 µL/s.

### Binding and selectivity measurements of the synthesized polymers

To determine the dissociation constants, 5 mg of the polymer (MIP or NIP) were mixed in an Eppendorf tube with 1 mL of IMD solution in 10 mM KH_2_PO_4_/K_2_HPO_4_ buffer (pH 7.2) at concentrations varying from 10 to 500 µM. The contact lasted 24 h under continuous agitation using unimax 1010 orbital shaker (Heidolph UK, Radleys) at 500 rpm. The solutions were transferred to Ultrafree—MC centrifugal filters (Milipore, Belgium) and centrifuged for 5 min at 4,500*g* using a benchtop centrifuge (Sigma 3-16 PK, SciQuip, Shrewsbury, UK). The remaining supernatant was evaporated until dryness at 40 °C using TurboVap LV (Biotage, Uppsala, Sweden), and the residue was reconstituted in 200 µL of the injection solvent. The remaining IMD concentrations were determined by LC–MS/MS (method described in the supplementary file). The amount of IMD bound to the polymers was calculated by subtracting the IMD concentration in the supernatant from the initial concentration of the standard solution. To analyze the ligand binding data to MIPs and NIPs, the Scatchard plot was used to determine the number of ligand binding sites and the affinities of each site. This method is widely used to evaluate the interaction between template molecules and MIPs/NIPs^10,41,42^. The Scatchard plot was applied by re-plotting the binding isotherm in the format of Q/C versus Q according to the equation:

1 $$ \frac{Q}{C} = \frac{{Q_{\max } - Q}}{{K_{{\text{d}}} }} $$

where Q is the amount of IMD bound to the polymers at equilibrium; C is the free IMD concentration at equilibrium; K_d_ is the dissociation constant and Q_max_ is the apparent maximum binding amount. The values of K_d_ and Q_max_ can be calculated from the slope and intercept of the linear line plotted in Q/C versus Q. Moreover, to verify the binding site selectivity the imprinting factor “*IF*” was calculated. The *IF* factor is best defined as the ratio of the distribution ratio for the analyte on the MIPs to the distribution ratio for the same analyte on the NIPs^43^. It can be calculated using the following equation:

2 $$ IF = \frac{QMIP/CMIP}{{Q{\text{NIP}}/CNIP}} $$

For the cross-reactivity and selectivity tests, 5 mg of the polymer (MIP or NIP) were mixed in an Eppendorf tube with 1 mL of different solutions of clothianidin, acetamiprid, thiamethoxam and thiacloprid in 10 mM KH_2_PO_4_/K_2_HPO_4_ buffer (pH = 7.2) each at a concentration of 10 µM. After 24 h of contact on an orbital shaker (500 rpm), the solutions were then centrifugated as previously described. The supernatants were then evaporated until dryness at 40 °C and the residue was reconstituted in 200 µL of the injection solvent. The remaining concentrations of the analytes were analyzed by LC–MS/MS (Table S2). Finally, the selectivity factor, α was calculated to compare between the selectivity of the synthesized MIPs towards IMD and to the other compounds^43^.

3 $$ \alpha = \frac{Qanalyte,MIP/Canalyte,MIP}{{Qcompetitor,{\text{MIP}}/Ccompetitor,MIP}} $$

## Results and discussion

### Characterization of the obtained particles

#### Scanning electron microscopy and dynamic light scattering measurements

**Figure 2:**
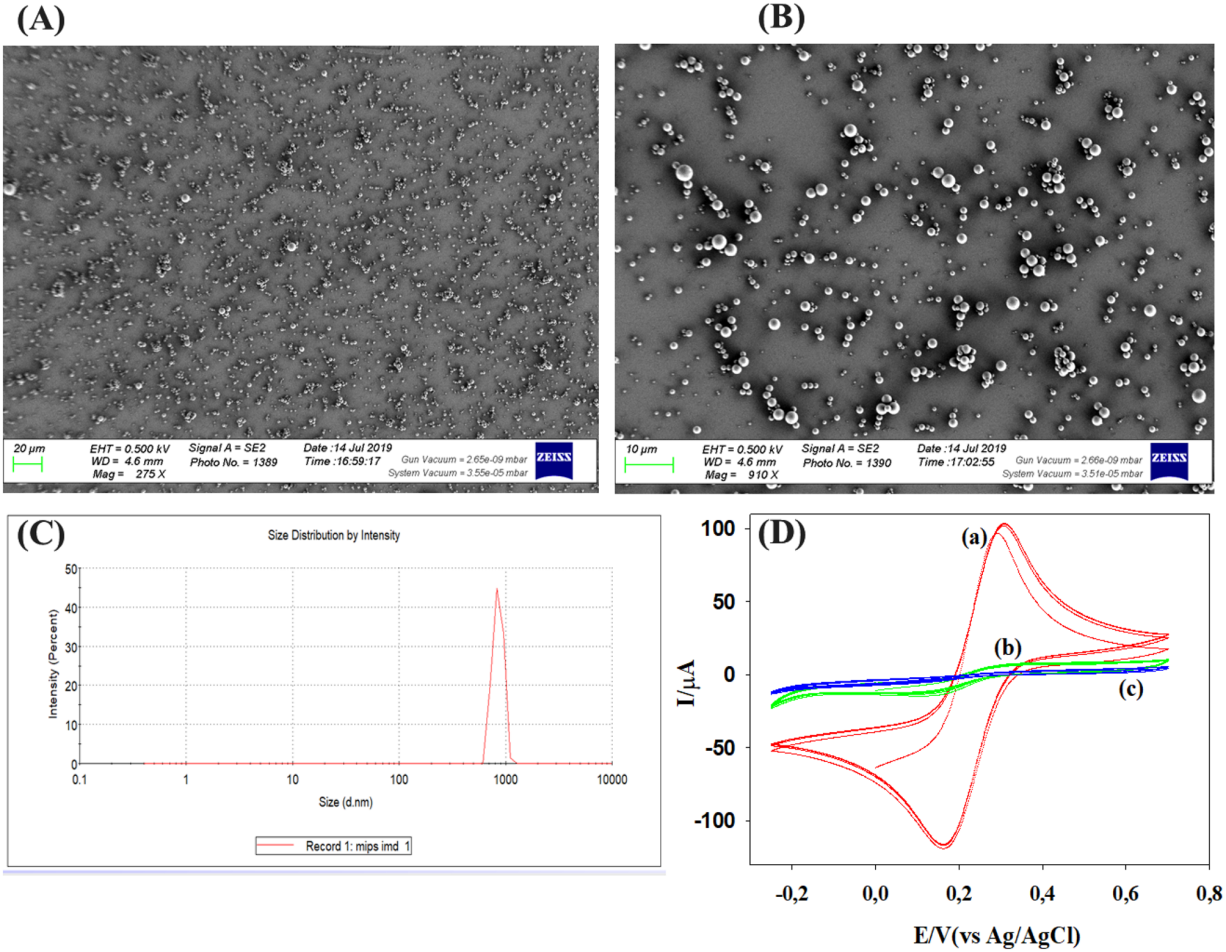
(**A**,**B**) Overview of SEM pictures of the electrode surface after the MIP-functionalization**. **(**C**) DLS measurement of the average particle size of the MIPs. (**D**) Cyclic voltammograms of a bare (a), MIP-modified (b) and 1-dodecanethiol end-capped gold electrode (c) in 10.0 mmol K_4_Fe(CN)_6_ and KCl electrolyte, at a scan rate of 100 mV.

The synthesized MIPs were suspended in the tyramine solution and electropolymerized on the gold electrode surface. Matrix entrapment was used to integrate those particles in the polytyramine layer on the electrode surface^44^. After coupling of the particles, the electrode surface was checked with scanning electron microscopy (SEM) as shown in Fig. 2A,B. The distribution of the particles on the gold electrode surface is clearly shown in these figures. Figure 2C shows the average particle size distribution [< 1 µm by dynamic light scattering (DLS)].

#### Cyclic voltammetry

Cyclic voltammetry was employed to check the surface of the gold electrode before and after immobilization of the MIPs, and after its blocking with 10 mM 1-dodecanethiol. Two pronounced peaks, one anodic and one cathodic, for the Fe (CN)_6_^3−^/Fe (CN)_6_^4−^ were observed for the bare gold electrode as shown in Fig. 2D.a. The redox peaks of the MIPs-modified electrode (Fig. 2D.b) were significantly smaller than for the bare gold electrode. This confirms that the immobilization was successfully done. Finally, Fig. 2D.c shows the electrode after being treated with 10 mM 1-dodecanethiol to block any bare site left on the electrode surface. The resulting curve has no redox current peaks which confirms that the electrode was successfully blocked and ready for the measurements.

### Binding properties of the MIPs and NIPs

**Figure 3:**
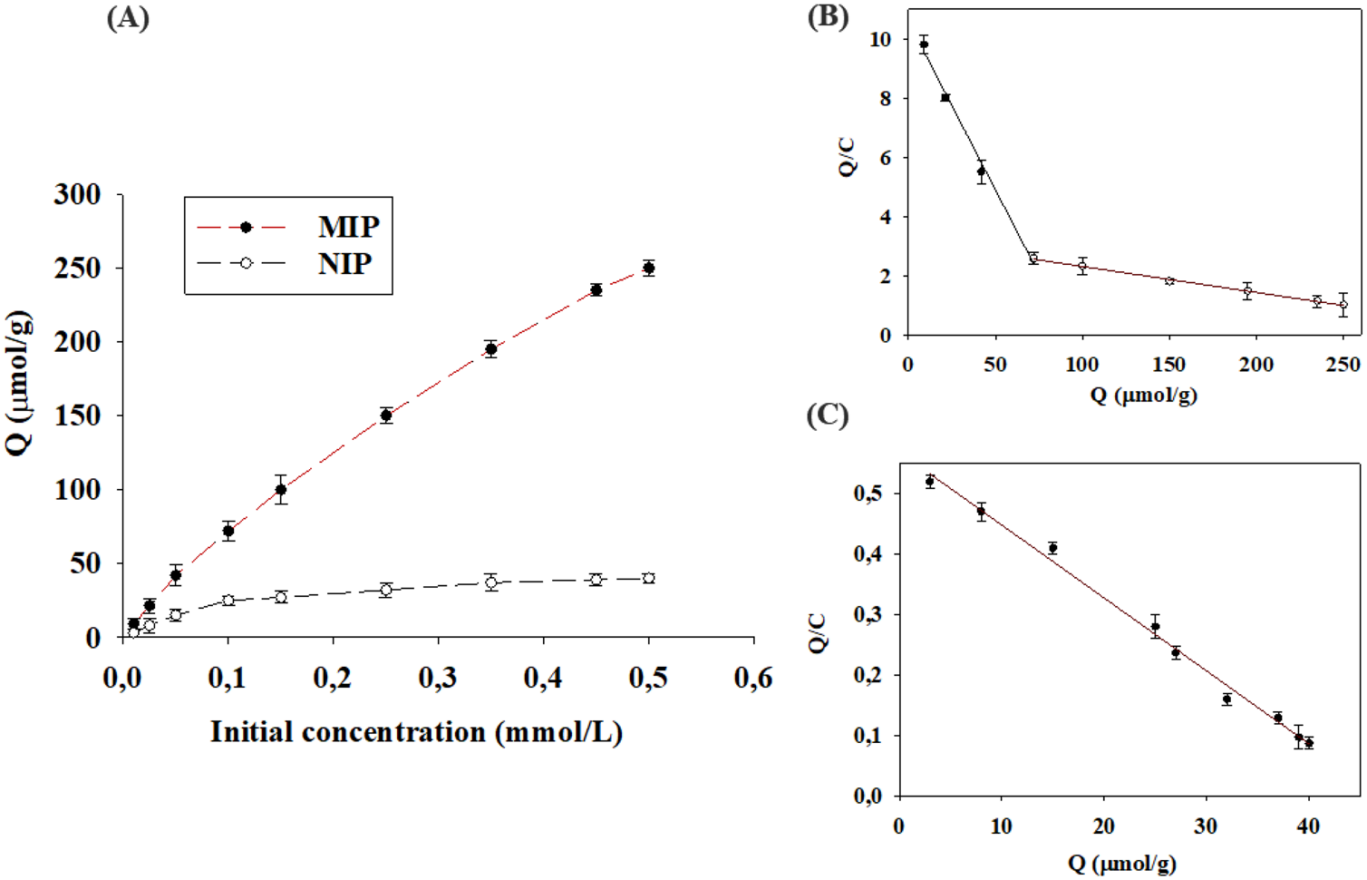
(**A**) Binding isotherm of IMD on the MIPs and the NIPs. IMD concentration: 10.0–500.0 µM; IMD volume: 1.0 mL; binding time: 24 h. (**B**) Scatchard plot analysis to estimate the binding nature of IMD onto the MIPs. (**C**) Scatchard plot analysis of the binding nature of IMD onto the NIPs. Q is the amount of IMD bound to 5.0 mg of MIPs or NIPs; C is the amount of free IMD in the solution.

As presented in Fig. 3A, the binding isotherm of IMD on the MIPs and NIPs shows that the amount of IMD bound to the MIPs was higher than that bound to NIPs which is due to the high affinity of the binding of the template to the polymer imprinted cavities. The Scatchard plot for the MIPs resulted into two linear parts with two sets of Q_max_ and K_d_ (Eq. 1). It suggests the presence of two classes of different binding sites. The linear regression equation for Fig. 3B.1 is Q/C = − 0.1138 Q + 10.577 (R^2^ = 0.9929) with K_d1_ = 8.78 µmol/L and Q_max1_ = 92.94 µmol/g. The linear regression equation for Fig. 3B.2 is Q/C = − 0.0088 Q + 3.1734 (R^2^ = 0.9963) with K_d2_ = 113.63 µmol/L and Q_max2_ = 360.6 µmol/g. By comparing the K_d_ from both lines, it confirms the presence of two classes of binding sites; one with a lower K_d_ value (K_d1_) attributed to the stronger binding site. The Scatchard method was also used to analyze the binding of IMD to the NIPs as presented in Fig. 3C and the linear regression equation was Q/C = − 0.0121 Q + 0.57 (R^2^ = 0.9913) with K_d_ = 82.62 µmol/L and Q_max_ = 47.10 µmol/g. This shows the presence of functional groups arranged randomly on the NIPs surface which can interact with the target analyte with a weaker binding. The existence of selectively imprinted sites on MIPs makes the binding stronger than to a NIP^43^.

### Cross-reactivity and selectivity of the MIPs and NIPs

**Figure 4:**
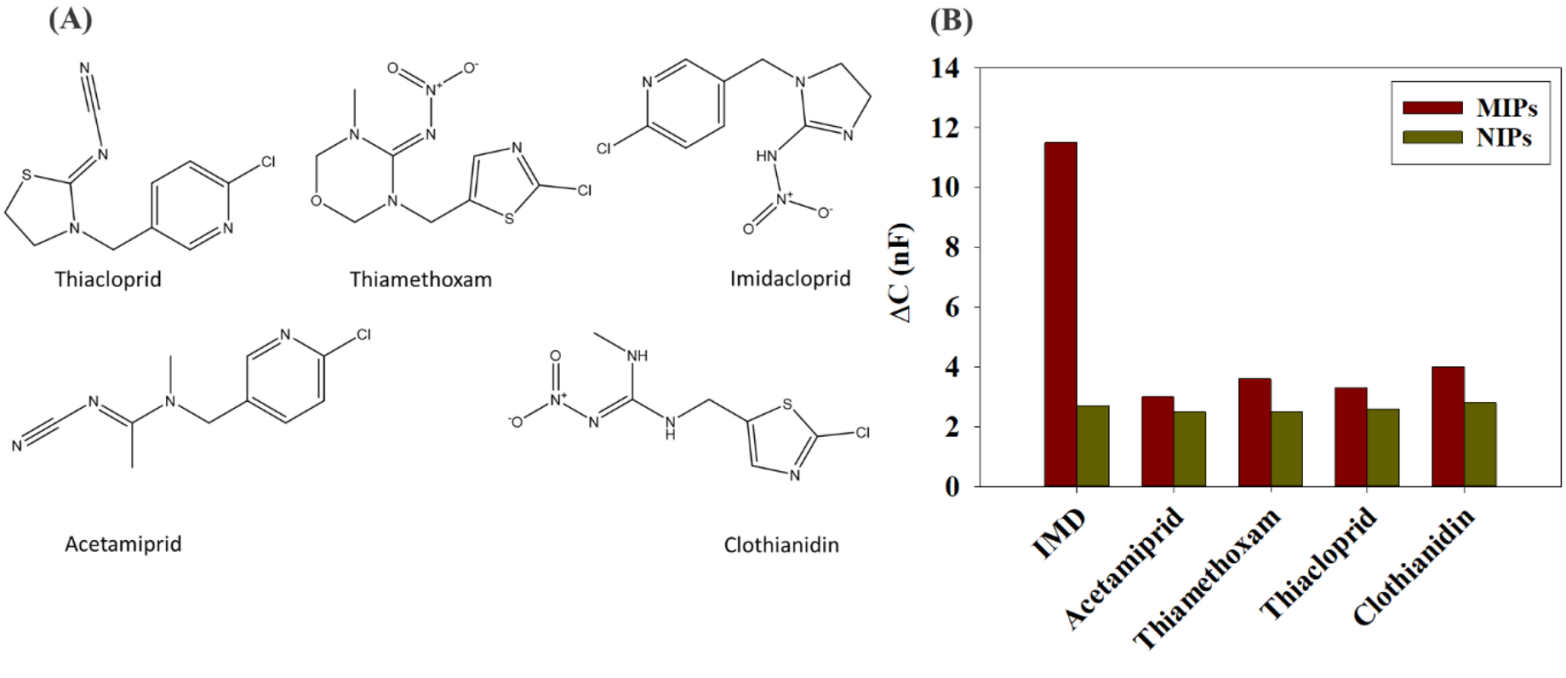
(**A**) Structural formulas of thiacloprid, thiamethoxam, imidacloprid, acetamiprid and clothianidin. (**B**) Differences between capacitance changes (nF) of the MIP and the NIP functionalized electrode in function of same concentration (100 µM) for separate injections of imidacloprid and other structurally similar neonics (acetamiprid, thiamethoxam, thiacloprid and clothianidin).

A cross-reactivity study was performed to compare the selectivity of the synthesized MIPs towards IMD with other structurally similar compounds (acetamiprid, clothianidin, thiamethoxam, thiacloprid) from the neonics class (Fig. 4A). The binding of the NIPs was also compared to the MIPs for each of them and for the main analyte (Fig. S1). By calculating the percentage bound to MIPs and NIPs for each compound, it was found that the difference between the binding to MIPs and NIPs for the competitors was not high compared to main analyte. This means there is no high selectivity of the synthesized MIPs to the other compounds. The *IF* factor for IMD was calculated using Eq. (2) and it was 6 for the synthesized MIPs. However, the *IF* factor is not a parameter to show the selectivity of the synthesized MIPs towards IMD as it does not show the binding to other compounds. Therefore, to compare to the other analytes, the selectivity factor (α) was calculated according to Eq. (3) for each compound to compare the binding of a competitor to the MIPs under the same conditions. As calculated and presented in Table 1, the values obtained for α were all greater than 1 which means that the particles are more selective to IMD than to the tested competitors. This study therefore shows that the synthesized MIPs exhibit a better selectivity and affinity towards IMD.

**Table 1:** Summary for the binding properties of the synthesized MIPs and NIPs.

K_d,MIP_	8.78 µmol/L, 113.63 µmol/L
Q_max,MIP_	92.94 µmol/g, 360.6 µmol/g
K_d,NIP_	82.62 µmol/L
Q_max,NIP_	47.10 µmol/g
IF_IMD_	6
α_acetamiprid_	14.8
α_thiamethoxam_	8.2
α_thiacloprid_	7.1
α_clothianidin_	6.8

### Capacitive detection of IMD

**Figure 5:**
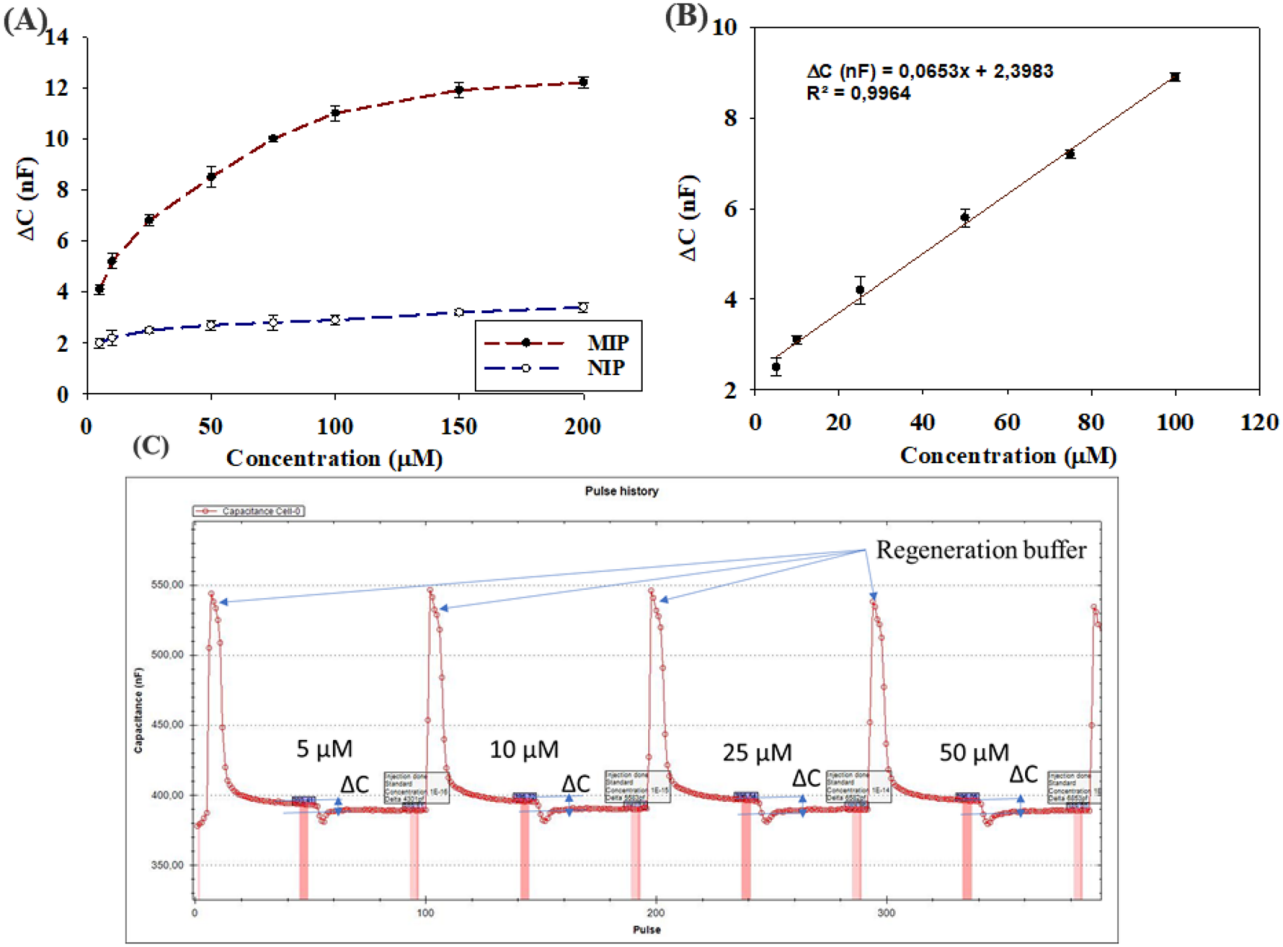
(**A**) Difference between capacitance changes in (nF) of the MIP and NIP functionalized electrode in function of IMD concentration (µM). The measurements were repeated three times with use of regeneration buffer between each injection. (**B**) Calibration curve plotted after subtraction of values of the NIP-functionalized electrode response**. **(**C**) Capacitance changes after injection of different concentrations of IMD for a MIP functionalized electrode under flow-injection analysis conditions with automated regeneration in between each injection.

IMD standards were prepared in ultra-pure water. In Fig. 5A, the change in capacitance is recorded as a function of different concentrations for both the MIPs- and NIPs-functionalized electrodes. The capacitive response for the MIP and the NIP was different. As shown in Fig. 5A, the drop in the capacitance values for the MIPs-functionalized electrode was more intensely pronounced than for the NIPs-functionalized electrode. The signal obtained from the MIPs- functionalized electrode was representative for both the specific and non-specific interactions. For the NIPs-functionalized electrode, the obtained signal was corresponding to the non-specific interactions. Therefore, the calibration curve was built after subtracting the NIP signal from the MIP signal to register only the specific interaction as shown in Fig. 5B. By calculating the change in capacitance as a function of IMD concentration, a regression line was obtained with a correlation coefficient (R^2^) of 0.9964 and a linear range of 5–100 µM. The equation of the regression line is ΔC = 0.0653 [IMD] + 2.3983. The response of the MIPs-functionalized electrode showed an LOD of 4.61 µM for the developed platform. As mentioned above, this class of pesticides is highly controversial, however there are few waters quality reference values for neonics in surface waters and the guidelines on water quality differ extensively by country while many are still under review. Comparing to other published MIP-based sensors in literature, the LOD ranged from 0.012 nM up to 10 µM for the detection of IMD in different matrices (vegetables^20^, fruits^45^, rice^31^ and juice^32^) which shows that our platform presents a good analytical performance. However, as we mentioned above, those sensors lack the re-usability and some of them show a difficulty in the template extraction and instability of the sensor on storage which is not suitable for on-site application.

### Validation of the sensor

The reproducibility, regeneration, repeatability and cross-reactivity were screened. The reproducibility was tested by measuring the same sample concentration with four different MIP-functionalized electrodes. The RSD values of the different electrodes tested were found to be in the range of (2.9–4.8%), showing the high reproducibility of the proposed technique. For at least 32 times, the electrode with the immobilized MIPs can be regenerated as shown in Fig. 5C and reused while maintaining a signal intensity of more than 90%. This is a necessary step in order to remove the bound analyte-MIP before the next measurement. Regeneration was performed using [MeOH/10 mM KH_2_PO_4_/K_2_HPO_4_ buffer (pH = 7.2)/triethylamine (47.5/47.5/5, v/v/v)] solution. This step is done spontaneously before each measurement as shown in Fig. 5C (also check Fig. S2). Therefore, we were able to test the repeatability by using the same electrode and same concentration. As mentioned above, each electrode maintained more than 90% signal intensity after 32 times of usage. Cross-reactivity experiments were performed with other neonics (acetamiprid, thiacloprid, thiamethoxam and clothianidin). Their concentrations were in the same order as for IMD (100 µM) to obtain a relevant comparison. The differences in capacitance changes generated by separate injection of these compounds are plotted in Fig. 4B, and the signal generated by IMD was at least three times higher than that of other tested compounds. Moreover, to evaluate the performance of the proposed MIP-chemosensor, the *IF* Factor and the selectivity factor (*α*) were calculated. The *IF* factor for the chemosensor is determined as the ratio of the slope of the calibration plot for the analyte using MIP to the slope of the calibration plot for the analyte using NIP. The selectivity factor (*α*) is determined as the ratio of the slope of the calibration plot for the analyte using MIP to the slope of the calibration plot for the interference using MIP. The calculated *IF* factor for IMD was 8.3 and the selectivity factor (*α*) for acetamiprid, clothianidin, thiacloprid and thiamethoxam were 7.3, 4.4, 5.3 and 5.8, respectively. This proves that the MIP-chemosensor exhibit better affinity and response towards IMD.

Finally, tap water (collected from Ghent, Belgium) and river water samples (collected from Giza, Egypt) were used to test the applicability of the developed sensor for IMD detection. Those samples were free from IMD as checked in advance by LC–MS/MS. The samples were used directly without any pretreatment steps by spiking them with a known concentration of IMD. The recovery percentages (n = 6) ranged from 94 to 106% which is acceptable for real application in environmental analysis.

## Conclusion

In conclusion, we developed a capacitive sensor based on MIPs for the selective determination of IMD in water. The MIPs were successfully synthesized by an easy and fast photoinitiated polymerization technique, washed and attached to the gold electrode surface by means of electro-polymerization. The particle size and shape were checked by DLS and SEM, respectively. The binding properties of the synthesized MIPs and NIPs were checked by LC–MS/MS. The obtained binding isotherm showed the presence of two classes of different binding sites on the MIP’s surface. The MIPs were able to bind more selectively to IMD than to structurally similar neonics. The *IF* factor and the selectivity factor *α* were determined to provide an evidence for the selectivity of MIPs to IMD. The proposed sensor showed a linear range of 5–100 µM with an LOD of 4.61 µM. The reproducibility and the number of regeneration cycles were also checked and showed high reproducibility and the possibility of reusing the same electrode up to 32 times due to the regeneration step. Finally, the proposed sensor was tested for environmental analysis by spiking tap and river water samples and it showed a relatively high recovery.

## Supplementary information

Supplementary information.
