# Supplementary material for: Capacitive sensor based on molecularly imprinted polymers for detection of the insecticide imidacloprid in water
Source: Sci Rep. 2020 Sep 2;10:14479. doi: 10.1038/s41598-020-71325-y (PMC7468110; doi:10.1038/s41598-020-71325-y)
Supplement: Supplementary file 1 — Supplementary information. [file 41598_2020_71325_MOESM1_ESM.docx]

***Capacitive sensor based on molecularly imprinted polymers for***

***detection of the insecticide imidacloprid in water***

Suzan El-Akaad^a,b*^, Mona A. Mohamed^b^, Nada S. Abdelwahab^c^, Eglal A. Abdelaleem^c^, Sarah De Saeger^a§^, Natalia Beloglazova^a,d,e§^

^a^Centre of Excellence in Mycotoxicology and Public Health, Faculty of Pharmaceutical Sciences, Ghent University, Ghent, Belgium

^b^Department of Pharmaceutical Chemistry, National Organization for Drug Control and Research (NODCAR), Giza, Egypt

^c^Department of Analytical Chemistry, Faculty of Pharmaceutical Sciences, Beni-suef University, Benisuef, Egypt

^d^Nanotechnology Education and Research Center, South Ural State University, Chelyabinsk, Russia

^e^Department of General and Inorganic Chemistry, Chemistry Institute, Saratov State University, Astrakhanskaya, Saratov, Russia

***Keywords:*** *Molecular imprinted polymer; imidacloprid; electrochemical detection; neonicotinoids; capacitive sensor.*

§= These co-authors contributed equally to this work

* Corresponding author: - [suzan.elakaad@gmail.com](mailto:suzan.elakaad@gmail.com) ; ORCID :0000-0002-5295-3810

**Table S1.** Summary of IMD- molecular imprinted polymers (application, preparation and parameters) available in literature.

| Application | Preparation method | Parameters | Reference |
| --- | --- | --- | --- |
| SPE- Chili and tomato | Bulk | 60 ̊C/ 24 h | [1] |
| SPE- Rice | Bulk | 60 ̊C/ 24 h | [2] |
| SPE- Eggplant and honey | Suspension | 60 ̊C/ 24 h | [3] |
| SPE- Water and soil | Bulk | 68 ̊C /24 h | [4] |
| Electrochemical- Celery juice | Surface | - | [5] |
| Electrochemical- Pears | Surface | - | [6] |
| Electrochemical-  Tomato, cabbage, chili and lettuce | Surface | - | [7] |
| Electrochemical- Rice | Surface | 60 ̊C / 24 h | [8] |
| Electrochemical- River and tap water | Emulsion | UV/ 1 h | This work |

**LC-MS/MS for neonics**

An Alliance HPLC 2695 chromatograph (Waters, Milford, MA, USA) apparatus coupled to a Micromass Quattro triple quadruple mass spectrometer (Waters, UK) equipped with a Z-spray electrospray ionisation (ESI) interface was used to assess the binding properties of the synthesized particles. Separation of IMD was achieved on an XBridge MS C18 column (150 mm× 2.1 mm I.D., 3.5 µm) with a guard column (10 mm× 2.1 mm I.D., 3.5 µm), both supplied by Waters. The mobile phase was a mixture of the eluents A [water/0.2 M ammonium bicarbonate (pH 10)/methanol (85/5/10, v/v/v) and B [water/0.2 M ammonium bicarbonate (pH 10)/methanol (5/5/90, v/v/v) at a flow rate of 0.15 mL min^-1^. A gradient elution was as follows: 0-1.5 min: 25-20% A; 1.5-2.7 min: 20-15% A; 2.7-4.2 min; 15% A; 4.2-4.8 min: 15-0.1%A; 4.8-5 min: 0.1% A; 5-5.5 min: 0.1-30% A; 5.5-7 min; 30% A. The column temperature was kept at 30 ^◦^C and the injection volume was 10 µL. The mass spectrometer was operated in the positive electrospray ionisation (ESI+) mode. MS parameters for the analysis were as follows: ESI source block and desolvation temperatures: 120 °C and 350 °C, respectively; capillary voltage: 3.2 kV; argon collision gas: 1.2×10-3 mbar; cone nitrogen and desolvation gas flows:100 and 830 l/h, respectively. MassLynx and QuanLynx software (Micromass, Manchester, UK) were used for the control of equipment, data acquisition and analysis. The data acquisition was performed in the multiple reaction monitoring (MRM) mode. The SRM transitions, the optimum cone voltages and collision energies selected for each transition are presented in Table S1. Each compound was injected separately since the method is not intended to target multiple neonics analysis.

**Table S2.** Optimized MS/MS parameters for the analysis of neonics in the SRM ESI + mode

| Neonics | RT, min | Precursor ion (m/z) | Cone voltage (*V*) | Product ions  (m/z) | Collision  energy (eV) |
| --- | --- | --- | --- | --- | --- |
| IMD | 3.3 | 256.1  [M+H]^+^ | 25 | 209 | 15 |
|  |  |  |  | 175 | 20 |
| Acetmiprid | 3.2 | 223.1  [M+H]^+^ | 28 | 125 | 20 |
|  |  |  |  | 187 | 15 |
| Clothianidin | 3.2 | 250.1  [M+H]^+^ | 22 | 132 | 15 |
|  |  |  |  | 169 | 15 |
| Thiamethoxam | 3.2 | 292.2  [M+H]^+^ | 22 | 211 | 15 |
|  |  |  |  | 181 | 25 |
| Thiacloprid | 3.3 | 253.1  [M+H]^+^ | 32 | 186 | 20 |
|  |  |  |  | 126 | 15 |

*Each compound was injected separately.

**Fig. S1** Differences between binding of the MIPs and NIPs to imidacloprid and 4 structurally similar neonics (acetamiprid, thiamethoxam, thiacloprid and clothianidin) expressed as bound percentage.


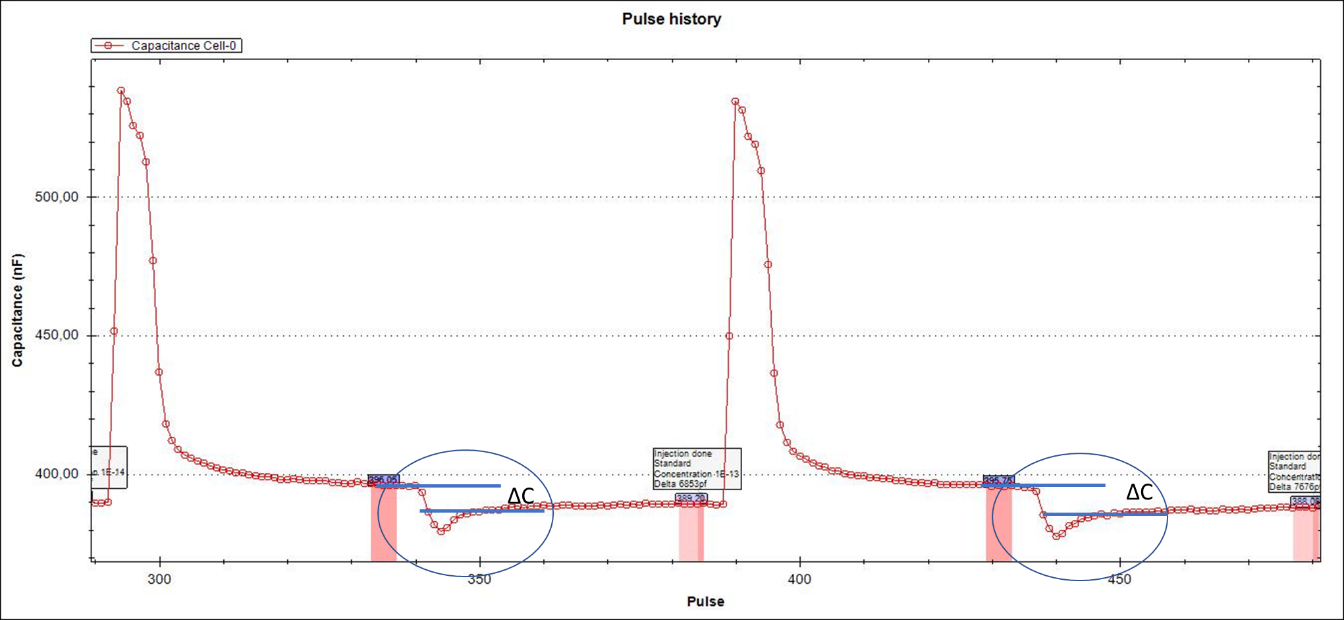


**Fig. S2.** A close zoom on the capacitance changes after injection of different concentrations of IMD for a MIP functionalized electrode under flow-injection analysis conditions with automated regeneration in between each injection.

1. Aria MM, Sorribes-Soriano A, Jafari MT, et al (2019) Uptake and translocation monitoring of imidacloprid to chili and tomato plants by molecularly imprinting extraction - ion mobility spectrometry. Microchem J 144:195–202

2. Chen L, Li B (2012) Determination of imidacloprid in rice by molecularly imprinted-matrix solid-phase dispersion with liquid chromatography tandem mass spectrometry. J Chromatogr B 897:32–36

3. Kumar N, Narayanan N, Gupta S (2018) Application of magnetic molecularly imprinted polymers for extraction of imidacloprid from eggplant and honey. Food Chem 255:81–88

4. Tang J, Zhang M, Cheng G, Lu Y (2009) Development and application of molecularly imprinted polymer as solid phase extraction of imidacloprid in environmental samples. J Liq Chromatogr Relat Technol 32:59–71

5. Bi X, Yang KL (2009) On-line monitoring imidacloprid and thiacloprid in celery juice using quartz crystal microbalance. Anal Chem 81:527–532

6. Kong L, Jiang X, Zeng Y, et al (2013) Molecularly imprinted sensor based on electropolmerized poly(o-phenylenediamine) membranes at reduced graphene oxide modified electrode for imidacloprid determination. Sensors Actuators, B Chem 185:424–431. https://doi.org/10.1016/j.snb.2013.05.033

7. Li S, Liu C, Yin G, et al (2016) Supramolecular imprinted electrochemical sensor for the neonicotinoid insecticide imidacloprid based on double amplification by Pt-In catalytic nanoparticles and a Bromophenol blue doped molecularly imprinted film. Microchim Acta 183:3101–3109

8. Zhang M, Zhao HT, Xie TJ, et al (2017) Molecularly imprinted polymer on graphene surface for selective and sensitive electrochemical sensing imidacloprid. Sensors Actuators, B Chem 252:991–1002
